# In Vitro Production of *Smilax brasiliensis* Seedlings, Callus Induction, Chemical Profile, and Assessment of Antioxidant Activity

**DOI:** 10.3390/plants14091383

**Published:** 2025-05-03

**Authors:** Paula Avelar Amado, Ana Hortência Fonsêca Castro, Lucas Santos Azevedo, Mariana Guerra de Aguilar, Lúcia Pinheiro Santos Pimenta, Luciana Alves Rodrigues dos Santos Lima

**Affiliations:** 1 Campus Centro-Oeste Dona Lindu, Universidade Federal de São João Del-Rei (UFSJ), Divinópolis 35501-296, MG, Brazil; paulaavelar28@yahoo.com.br (P.A.A.); acastro@ufsj.edu.br (A.H.F.C.); azevedolucas07@gmail.com (L.S.A.); 2 Departamento de Química, Instituto de Ciências Exatas, Universidade Federal de Minas Gerais (UFMG), Belo Horizonte 31270-901, MG, Brazil; marianag.a9@gmail.com (M.G.d.A.); lpimenta@ufmg.br (L.P.S.P.)

**Keywords:** germination, acclimatization, plant tissue culture, phenolic compounds, free radicals, ^1^H NMR

## Abstract

This study aimed to assess the production of *Smilax brasiliensis* seedlings in an in vitro environment and their adaptation to natural conditions, as well as the callus induction, the chemical profile of calli extracts, and their antioxidant potential. The seedlings were obtained from *S. brasiliensis* seeds germinated in Murashige and Skoog (MS) medium. The germination rate was 33%, and about 22% of the seeds produced whole seedlings. Three-month-old seedlings were acclimatized for two months, resulting in an 80% survival rate and improved physiological characteristics. Callus induction was initiated from leaf explants obtained from seedlings and plant growth regulators (PGRs), with and without light exposure. Calli extracts were obtained using methanol; phenolic compound and flavonoid quantification were performed, and the chemical profile was determined by nuclear magnetic resonance (^1^H NMR). For comparison, methanol extract from *S. brasiliensis* leaves collected in Brazilian Cerrado were also analyzed. Antioxidant activity was assessed using the 2,2-diphenyl-1-picryl-hydrazyl method and the ferric-reducing antioxidant power assay. All samples exhibited antioxidant activity according to the methods employed. Furthermore, ^1^H NMR revealed metabolic profile changes in the calli extracts compared to the leaf extract. This study yielded promising results, suggesting that in vitro culture could improve productivity and conserve the species, although changes were observed in the metabolic profile of *S. brasiliensis*.

## 1. Introduction

*Smilax brasiliensis* Sprengel (Smilacaceae), popularly known as “japecanga” or “sarsaparilha”, is native to the Cerrado, a biome that occupies 25% of Brazilian territory [1,2]. It is widely distributed in the Midwest (Federal District, Goiás, Mato Grosso do Sul, Mato Grosso) and Southeast (Minas Gerais, São Paulo) regions of Brazil [3]. This species is a climbing plant, characterized by oval, leathery leaves, with three central veins and spines only on the central vein of the abaxial surface; stems with several spines [4]. The flowers are greenish, the berry globose, green to wine-purplish and black. The seeds are reddish [5]. *S. brasiliensis* is used in folk medicine to treat inflammation, skin infections, and sexually transmitted diseases [1,2,3,4,5,6]. These effects are reported for leaves and roots, which could be related to phenolic acids, flavonoids, triterpenoids, coumarins, saponins, and steroids [7,8]. The antioxidant activity and absence of cytotoxic effects of petroleum ether and methanol extracts, fatty acids, and methyl esters from *S. brasiliensis* leaves were demonstrated for the first time by Amado et al. [7]. Similarly, Amado et al. [9] showed the larvicidal activity of phenolic compounds present in extracts and fractions obtained from *S. brasiliensis* leaves against *Culex quinquefasciatus* and confirmed their antioxidant activity and absence of cytotoxicity. Some studies have also demonstrated the allelopathic and phytotoxic effects of leaf extracts and fractions, indicating that *S. brasiliensis* can be used as a natural herbicide [8,10,11]. Recently, Silva et al. [12] showed that *S. brasiliensis* stems are also a rich source of polyphenol compounds, exhibiting high antioxidant potential without toxicity, and that they can be used in food supplements or as natural antioxidants in the food industry. Therefore, these findings make *S. brasiliensis* a promising species for medicinal and agricultural uses.

In nature, *Smilax* sp. regenerates through seeds, which are only produced during a few months of the year (August–November), and the establishment of seedlings is slow, hampered by unfavorable environmental conditions, and pathogen and herbivore attacks. The seeds exhibit low germination rates due to integumentary dormancy [13]. Seed germination is considered one of the most critical steps for successful seedling establishment and efficient plant growth and development. Plant tissue culture is a useful tool for providing strategies for improving crop growth [14]. Micropropagation enables the production of healthy seedlings in a controlled and aseptic environment [15]. The success of micropropagation depends on effective acclimatization strategies that guarantee the survival of seedlings during the transfer from in vitro to ex vitro conditions, where there is high light intensity, excessive water loss, and changes from heterotrophic metabolism to autotrophic metabolism [16].

Callus is an undifferentiated tissue that multiplies uncontrollably and develops from plant tissues by culture in solid medium that usually contains balanced amounts of plant growth regulators (PGRs), to maintain the undifferentiated stage [17,18]. Callus culture is a biotechnological tool that can be used to produce bioactive compounds of interest in a sustainable and renewable way because it allows the modulation of plant metabolite synthesis, with continuous production occurring in a controlled environment, in a smaller space, and in less time [19,20].

So far, there are no reports on the in vitro culture of this species. So, in the current study, we describe the in vitro germination and seedlings acclimatization, as well as the callus induction, the chemical profile analysis, quantification of phenolic and flavonoid compounds, and the assessment of the antioxidant activity of the extracts obtained from callus induced by different treatments with PGRs from leaf explants of the *Smilax brasiliensis*.

## 2. Results

### 2.1. In Vitro Germination

Germination was evaluated based on root protrusion. The seeds germinated after 17 days of inoculation. The observed germination rate was 33%, and the growth rate of whole seedlings (with roots and leaves) was 22%. There was no contamination and/or oxidation in the seeds and in the culture medium during the germination, demonstrating the efficiency of the asepsis process and the absence of release of phenolic compounds in the medium during seed scarification, respectively.

### 2.2. Seedling Acclimatization

Seedlings transferred to an ex vitro environment consisting of plastic cups containing vermiculite showed an average stem length of 7.0 ± 1.0 cm, an average main root length of 3.9 ± 0.6 cm, and a number of leaves and roots equal to 10.0 ± 2.1 and 9.6 ± 3.4, respectively, on average. After the acclimatization process, the seedlings had a stem length equal to 13.0 ± 1.1 cm, a main root length equal to 7.0 ± 2.0 cm, a number of leaves equal to 14.2 ± 3.1, and a number of roots equal to 16.0 ± 4.9 on average, showing an increase in all morphological characteristics observed. The survival rate of seedlings was 80%, demonstrating that the use of vermiculite associated with ex vitro culture conditions favored the acclimatization of seedlings. These results showed the success of the acclimatization process and the possibility of obtaining *Smilax brasiliensis* seedlings adapted to the ex vitro environment through in vitro seed germination, which enables species conservation.

### 2.3. Callus Induction

The conditions used for callus induction are shown in Table 1.

Callus induction was only observed in T4 in the presence of light and in T1, T2, and T6 in the absence of light after 5 months. No callus induction occurred in the absence of PGRs. There was no contamination and/or oxidation in the callus or the culture medium. The induction percentage, consistency, fresh and dry mass, and dry mass yield of the calli are shown in Table 2.

The calli showed greater fresh mass production in T4 and T6, but after drying, the best yield was observed in T2, whose calli had a lower amount of water and, consequently, greater mass, with a yield of 30.05% of dry mass. All treatments induced yellow friable calli.

### 2.4. Obtaining Extracts

The dry calli and *S. brasiliensis* leaves (1 g) collected in the Brazilian Cerrado were extracted with methanol (10 mL) using acoustic cavitation. The extraction process was repeated two more times. At the end of the process, the extracts obtained in the three extractions of each treatment were combined.

The masses and yields for each extraction obtained from the dry calli in different treatments and leaf methanol extract (ME) are shown in Table 3. The yields were calculated in relation to 1 g of dry callus or leaves used in the extraction.

Regarding the extraction methodology, there was a higher yield in the first stage compared to the other steps, but it was essential to perform three extractions to increase the final yield. Treatment 1 showed a better yield after the three extractions, which was around 25.49% compared to the dry callus and leaf mass used in the extraction.

### 2.5. Determination of the Metabolic Profile of Methanol Extracts by Nuclear Magnetic Resonance (NMR)

Several analytical techniques, including nuclear magnetic resonance (NMR), mass spectrometry (MS), and high-performance liquid chromatography (HPLC), have been utilized to determine the chemical profile of various biological systems. NMR stands out in this field due to its unique advantages, such as speed, accuracy, simultaneous detection of compounds, nondestructive nature, repeatability, and straightforward sample preparation [21,22]. One of the significant benefits of NMR is its ability to provide chemical information about complex mixtures, allowing for the identification and semi-quantification of certain metabolites without prior separation. In this study, NMR was employed to assess changes in the metabolic profile of *S. brasiliensis* calli grown under different conditions and, when possible, classify the primary and secondary metabolites present in the samples.

**Figure 1:**
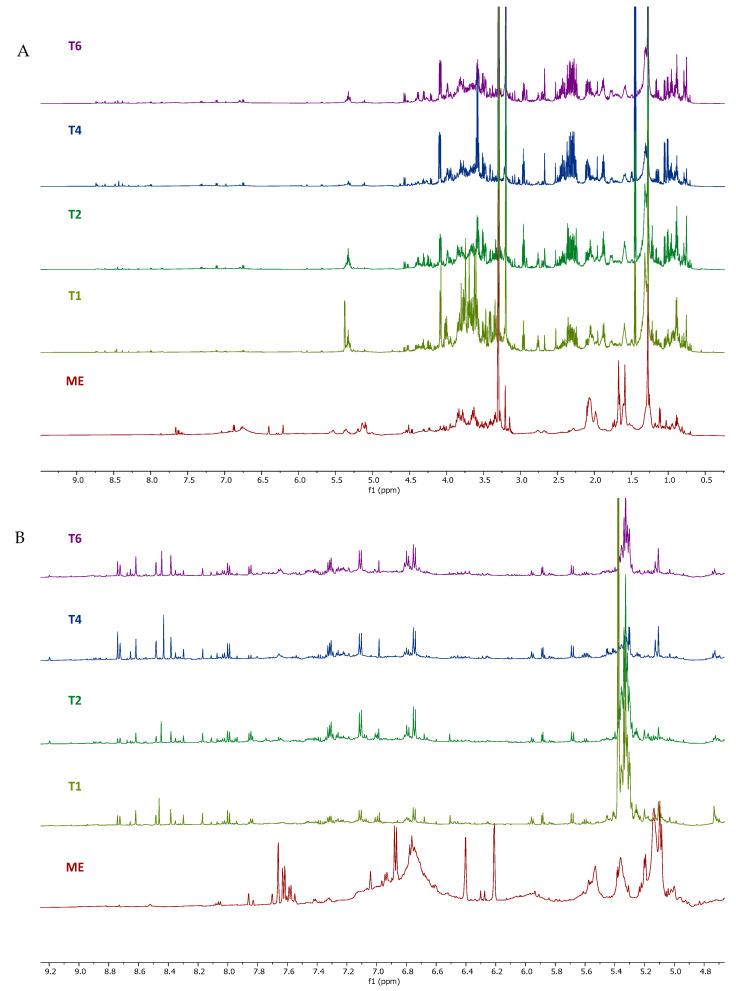
^1^H NMR spectra (600 MHz, methanol-*d*_4_ containing 0.01% (*w*/*v*) TSP-*d*_4_) of the calli methanol extracts from *Smilax brasiliensis* and the leaf methanol extract (**A**); (**B**) Expansion in range of 4.8–9.20 ppm. T6: Treatment 6 (0.5 μg/mL picloram + 0.5 μg/mL BAP), T4: Treatment 4 (1.0 μg/mL picloram), T2: Treatment 2 (1 μg/mL 2.4-D), T1: Treatment 1 (0.5 μg/mL 2.4-D), ME: Leaf methanol extract.

**Figure 2:**
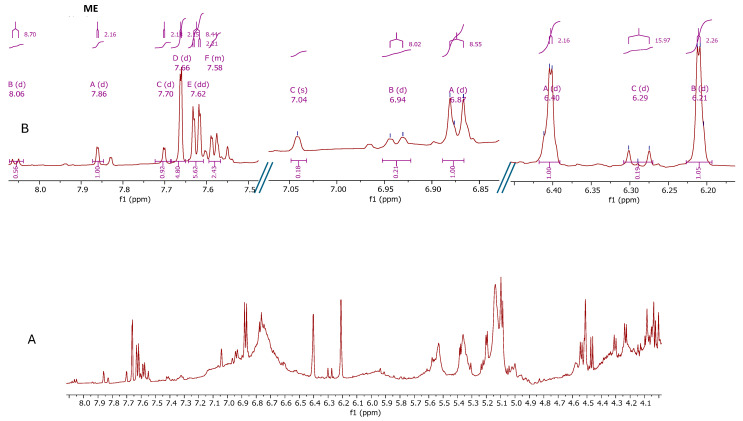
^1^H NMR spectra (600 MHz, methanol-*d*_4_ containing 0.01% (*w*/*v*) TSP-*d*_4_) of (**A**) leaf methanol extract from *Smilax brasiliensis* in the range of 4.0–8.1 ppm; (**B**) Expansion in aromatic region in a range of 6.20–8.05 ppm.

**Figure 3:**
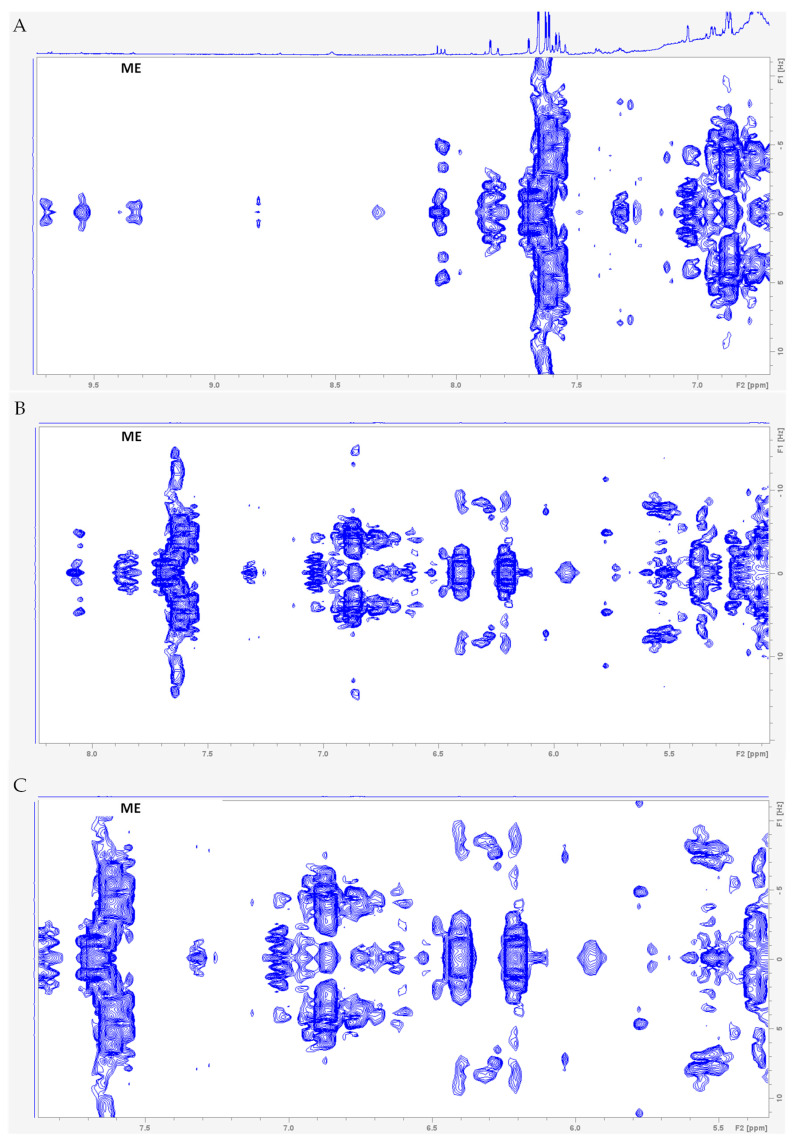
*J*−resolved nuclear magnetic resonance (NMR) spectra (600 MHz, methanol-*d*_4_ containing 0.01% (*w*/*v*) TSP-*d*_4_) of the leaf methanol extract from *Smilax brasiliensis*, in the region of 9.7–6.7 ppm (**A**), 8.2–5.0 ppm (**B**), and 7.9–5.3 ppm (**C**), respectively.

The metabolic profile of the calli methanol extracts from *S. brasiliensis* obtained from different treatments was analyzed using ^1^H NMR spectra and compared to the spectra of the leaf methanol extract obtained from *S. brasiliensis* collected from Brazilian Cerrado. The calli methanol extracts showed a distinct metabolic profile compared to the leaf. The ^1^H NMR spectra were analyzed by focusing on three regions: *δ* 10-6, *δ* 6-3, and *δ* 3 to 0. The calli extracts presented different and more intense signals than the leaf extract in the aromatic region, specifically in *δ* 10-8 (Figure 1 and Figure 2). Although the use of NMR in metabolic profiles has many advantages, the overlapping of the signals in the NMR spectra represents a major difficulty in metabolite identification. This problem can be minimized using the housing hold database and obtaining 2D ^1^H-^1^H COSY and *J*-Resolved spectra (Figure 3). However, in the aromatic region, the low signal intensity and lack of reference compound spectra were the main obstacles to compound identification.

The 1D and 2D NMR spectra of the leaf methanol extract were analyzed, and some compounds were putatively characterized (Figure 2). The aromatic region showed the presence of one doublet in *δ* 6.29 (*J* = 15.97 Hz) and signals the region of *δ* 7.56–7.66, which are typical signals of H-8′ and H-7′ in phenylpropanoids, respectively [21,23] (Figure 2B). The COSY spectra confirmed this correlation (Figure 3). These signals were compatible with the presence of chlorogenic acid, caffeic, and feruloyl derivatives. Additionally, quercetin and kaempferol flavonoid glycosides were identified. Quercetin in glycosylated form were identified with the following signals: *δ* 7.62 (*dd*, *J* = 8.46, 2.19 Hz), 7.58 (*d*, *J* = 8.46 Hz), 6.87 (*d*, *J* = 8.46 Hz), 6.40 (*d*, *J* = 2.17 Hz), and 6.21 (*d*, *J* = 2.17 Hz). Kaempferol derivatives were identified with the signals *δ* 8.06 (*d*, *J* = 8.66 Hz) and 6.88 (*d*, *J* = 8.66 Hz) [21,23] (Figure 2B). These compounds, characterized in the leaf methanol extract of *S. brasiliensis* [9,11], were barely observed in the calli spectra, suggesting that their biosynthesis was modified in the growth conditions.

**Figure 4:**
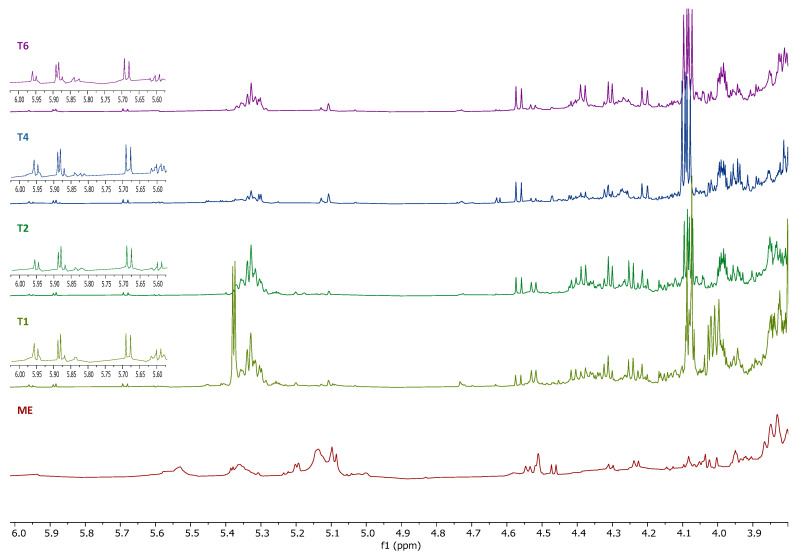
^1^H NMR spectra (600 MHz, methanol-*d*_4_ containing 0.01% (*w*/*v*) TSP-*d*_4_) of the calli methanol extracts from *Smilax brasiliensis* and the leaf methanol extract, expansion between 3.8–6.0 ppm. T6: Treatment 6 (0.5 μg/mL picloram + 0.5 μg/mL BAP), T4: Treatment 4 (1.0 μg/mL picloram), T2: Treatment 2 (1 μg/mL 2.4-D), T1: Treatment 1 (0.5 μg/mL 2.4-D), ME: Leaf methanol extract.

The region *δ* 4.0–5.5 was the carbohydrate region and the region of olefinic signals of unsaturated fatty acid. The leaf methanol extract seemed to produce different carbohydrate compounds as well as unsaturated fatty acids (Figure 4) compared to the calli. The anomeric protons of *α*-glucose (*δ* 5.19), *β*-glucose (*δ* 4.57), *α*-rhamnose (*δ* 5.09; 1.17), *β*-rhamnose (*δ* 5.04; 1.12), *L*-fucose (*δ* 4.23), fructose (*δ* 4.14), and sucrose (*δ* 5.38; 4.14) were detected in the leaf methanol extract. Doublets in *δ* 5.95, 5.88, 5.68, 5.60, and 5.38 (T1) [21,23,24] were observable in calli extracts but did not appear in the leaf methanol extract. The doublet at *δ* 5.38 can be assigned to sucrose and was observed only in T1. The other doublets present in calli extracts might be from the PGR added to the medium.

**Figure 5:**
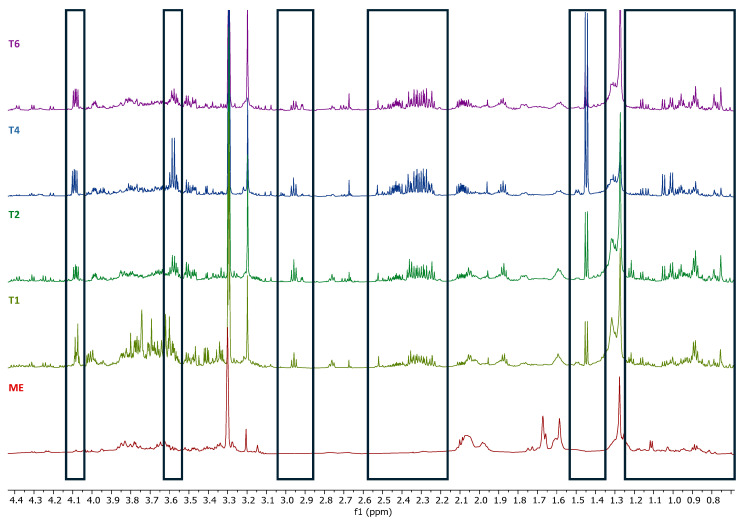
^1^H NMR spectra (600 MHz, methanol-*d*_4_ containing 0.01% (*w*/*v*) TSP-*d*_4_) of the calli methanol extract from *Smilax brasiliensis* and the leaf methanol extract, expansion between 0.7–4.4 ppm. T6: Treatment 6 (0.5 μg/mL picloram + 0.5 μg/mL BAP), T4: Treatment 4 (1.0 μg/mL picloram), T2: Treatment 2 (1 μg/mL 2.4-D), T1: Treatment 1 (0.5 μg/mL 2.4-D), ME: Leaf methanol extract. The boxes highlight the main differences among the calli extracts and the leaf extract.

Additionally, in the leaf methanol extract, signals between *δ* 5.52 and 5.35 coupled with methinic and methylene signals in the range of *δ* 1.72–2.14 were characteristic of unsaturated fatty acids, whose *ω*-methyl group showed a signal at *δ* 0.98. The area between *δ* 0.8 and 3, with intense signals observed in the calli extracts, is characteristic of amino acids and organic acids [24], as well as terpenes. An intense doublet signal at *δ* 1.44 was characteristic of calli extracts and absent in the leaf extract, and may be associated with the methyl group of lactate. The signal of the lactate methinic group was also observed at *δ* 4.1 [24]. A triplet in *δ* 2.95 was also observable only in the calli spectra (Figure 5).

The more intense signals in the region of *δ* 3.21-0.75 (Figure 5) suggest the higher contents of lipophilic compounds, such as unsaturated common fatty acids, triterpenoid or steroid compounds, and amino acids in the calli extracts that were not present in the leaf extract. The *Smilax* genus is known for possessing saponins [25] which can be triterpene or steroidal, and the signals in HSQC spectra (Figure S1, Supplementary Materials) at *δ* 0.77/15.24, 0.77/19.45, 0.78/18.75, 0.92/23.20, 0.96/16.84, 1.00/19.69, 0.91/23.20, 1.12/18.28, 1.16/21.33, and 1.44/19.22 are characteristics of methyl groups described in these compounds in the literature [26,27]. In addition, two doublets at *δ* 4.38 and 4.29 coupled with carbon signals at *δ* 104.31 and 107.12 suggested the presence of glycosides or terminal methylene protons of exocyclic double bonds, reinforcing the presence of triterpene/steroidal saponins in the calli [26,27,28]. COSY spectra (Figure S2, Supplementary Materials) confirm these assignments and show that there might be unsaturation in the steroidal ring by coupling the signals at *δ* 5.33 with *δ* 2.04. The presence of unsaturated fatty acids was putatively assigned by the signals in the HSQC spectra *δ* 5.29/131.03 and 5.32/132.9, and the coupling of the signals *δ* 5.29/2.81, 5.29/2.78, and 5.32/3.03 in the COSY spectra [24].

**Figure 6:**
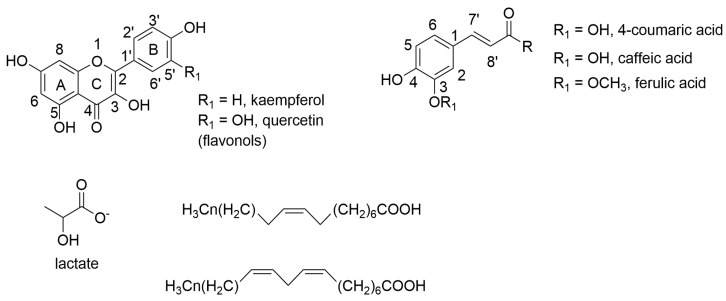
Structures of some compounds found in the methanol extracts.

The structures of some compounds present in the methanol extracts are shown in Figure 6.

### 2.6. Determination of the Total Phenolic Compound, Flavonoid Content, and Antioxidant Activity

The total phenolic compound and flavonoid contents were also quantified in the samples. The leaf methanol extract showed higher total phenolic compounds and flavonoid contents than the extracts obtained from calli samples. For total phenolic compounds, there were no great differences among the calli samples, but the highest levels were observed for calli from treatments 2 and 4. As for total flavonoids, the highest contents were detected in calli from treatments 1 and 2 (Table 4).

The results for the antioxidant activity obtained by 2,2-diphenyl-1-picryl-hydrazyl (DPPH) and ferric-reducing antioxidant power (FRAP) assays are shown in Table 4 and Table 5.

As shown in Table 5, all samples exhibited antioxidant activity using the DPPH and FRAP methods. In the DPPH method, the samples showed better activity than the 2,6-di-*tert*-butyl-4-methylphenol (BHT, control) at concentrations of 1 and 10 μg/mL. The leaf methanol extract also exhibited greater DPPH free radical capture than BHT at 100, 250, and 500 μg/mL (Table 5). In the FRAP test, the leaf methanol extract also presented more significant antioxidant potential than BHT and other samples. For the extracts obtained from calli samples, treatments 4 and 6 demonstrated better antioxidant activity using the DPPH and FRAP methods (Table 4).

## 3. Discussion

This is the first report on the in vitro germination, seedling acclimatization, and callus induction of *Smilax brasiliensis* as well as the determination of metabolic profiles by ^1^H NMR, and assessment of antioxidant activity of calli culture. Studies related to the in vitro propagation of *Smilax* spp. and the production of secondary metabolites are scarce, especially through in vitro germination and callus induction.

Propagation studies generally aim to conserve species. Most ex situ plant biodiversity conservation strategies have focused on agricultural species [29]. However, the improvement of conservation techniques for plants with medicinal potential has also become the target of important studies [30,31]. For medicinal species, in vitro culture is a traditional conservation strategy, with specific techniques for the collection, multiplication, and storage of plant germplasm, representing a good option for the safe conservation of species of pharmacological interest [30,32,33].

This work on the propagation of *S. brasiliensis* presented a method for its sustainable management. In the present study, the seeds germinated 17 days after inoculation was set up, with a germination percentage of 33%. Although *Smilax* spp. seeds present integumentary dormancy [13], this was overcome mechanically in our study using a scalpel. Optimization of culture conditions through seed disinfection with 25% sodium hypochlorite for 15 min, associated with mechanical scarification of seeds, and seed imbibition in autoclaved distilled water for 2 h, contributed to improving the observed germination and survival rate. The *Smilax* spp. seeds are very demanding since germination occurs only after many weeks to months under imbibition in a narrow range of optimal temperatures, and there is a rapid loss of viability if the seeds are imbibed in suboptimal temperatures [34,35,36]. Some studies report that the germination of *S. brasiliensis* seeds is influenced by both light and temperature, with the highest germination rates (55%) and more rapid germination (germination speed index = 1.57) being reached under conditions of 20–30 °C with the absence of light [36,37]. This fact may explain the lower germination percentage achieved in vitro, since the seeds germinated in the presence of light (16:8 h light/dark regime, light intensity of 40 μmol/m^2^ s^1^).

One of the main challenges of in vitro seedling production is finding methods to improve the survival rate and tissue growth parameters during acclimatization. The seedlings are susceptible to various stressors typical of the ex vitro environment, and they need to change from heterotrophic to autotrophic feeding [38]. In addition, geoclimatic and seasonal changes and external temperature and humidity conditions negatively affect many plant metabolic processes [39]. Seedlings from in vitro culture have thin cuticles, non-functional stomata, and a poorly developed root system [40], which can affect survival or result in the formation of non-viable seedlings [38]. However, these problems can be controlled using special culture conditions in an ex vitro environment. Therefore, for the conservation and sustainable management of *S. brasiliensis*, knowledge about adaptation in a new environment is necessary, but studies on the acclimatization of *Smilax* spp. are still scarce. For *Smilax nageliana*, a native plant from Indonesia, the acclimatization process was evaluated under three treatments: (i) hood application in a greenhouse; (ii) its natural habitat under shade; and (iii) natural habitat in an open area. *S. nageliana* showed a 100% survival rate in the hood application and natural habitat with shade [41]. *Smilax corbularia* seedlings were acclimatized in plastic pots containing soil, carbonized rice, hull, decomposed rain tree leaves, manure, and sand in a ratio of 0.5:0.5:0.5:1:1, obtaining a survival rate of 80.81% after 4 weeks of seedling transfer [42]. No reports have been found in the literature on the acclimatization of *S. brasiliensis*.

Plant tissue culture techniques enable high efficiency and quick production of aseptic and pathogen-free plants and provoke considerable interest as a potential alternative to produce bioactive compounds [43]. The present study describes callus induction in *S. brasiliensis* from leaf explants cultivated in Murashige and Skoog (MS) medium [44] supplemented with different concentrations of dichlorophenoxyacetic acid (2,4-D), picloram, and 6-benzylaminopurine (BAP). There was no oxidation in the callus and/or in the culture medium, which is common in the in vitro culture due to the release of phenolic compounds from the explant. Calli induced in the presence of 1 μg/mL 2.4-D, and in the absence of light, showed a higher dry matter yield (Table 2), an important result when considering the mass obtained by the preparation of plant extracts from callus. Auxin 2,4-D is recognized for its callus-inducing effect on most plants and has been widely used either alone or combined with cytokinins to stimulate callus induction and obtain bioactive compounds in vitro. However, the concentration and combination of PGR must be defined for each species [45]. Some studies have demonstrated the effect of certain auxins on callus induction in *Smilax* spp. Kumar et al. [43] showed that MS medium supplemented with individual growth regulators was not satisfactory for obtaining callus. However, when these regulators were combined, callus formation was observed in *Smilax wightii* leaf explants, in which the best result was for the medium containing 1.5 mg/L thidiazuron (TDZ) and 0.02 mg/L 1-naphthaleneacetic acid (NAA).

This study obtained methanol extracts using acoustic cavitation, with total yields ranging from 9.78% to 25.49%. Previous studies with *S. brasiliensis* using different extraction methods have already been reported. Amado et al. [9] obtained the methanol extract of the leaves using a Soxhlet extractor, with a yield of 10.94%. Silva et al. [12] extracted the dried stems by percolation with ethanol, producing the ethanol extract (yield of 6.12%). Thus, we observed that the acoustic cavitation used in this work provided a higher yield than extraction by Soxhlet and percolation. Acoustic cavitation is a method based on the cavitation effect generated by ultrasonic waves to increase the penetration of solvents into plant material, allowing efficient extraction of bioactive compounds, and improving the quality and yield of the extract [46].

The quantification of total phenols and flavonoids in calli extracts demonstrated lower levels compared to the leaf methanol extract, as shown in Table 4. The NMR spectra suggest that the different treatments with PGR may influence calli’s metabolic pathway, changing metabolism in relation to leaves of *S. brasiliensis.* The chemical profiles of the calli extracts obtained by ^1^H NMR spectra suggest the presence of lipophilic compounds, such as unsaturated common fatty acids, triterpenoids, and steroid compounds, which corroborate the results observed for the content of phenolic compounds and flavonoids. Total phenol and flavonoid contents in lipophilic extracts for *S. brasiliensis* were evaluated by Fonseca et al. [10] and Silva et al. [12]. The total phenols and flavonoids for the hexane extract of *S. brasiliensis* leaves were 64.12 μg GAE/mg and 23.41 μg QE/mg, respectively [10]. The hexane fraction of *S. brasiliensis* stems demonstrated a total phenol and flavonoid content of 31.60 μg GAE/mg and 20.18 μg QE/mg, respectively [12]. Similar results were found in this study for calli extracts in relation to total phenols and flavonoids.

The antioxidant activity of *S. brasiliensis* calli extracts was evaluated using the DPPH and FRAP methods. The results of this study corroborate the values found for the hexane fraction in the stems by Silva et al. [12], with an IC_50_ value of 161.75 μg/mL for the DPPH assay and IC_50_ = 10.35 μg/mL for the FRAP assay.

The similarity of the results found in this study with those of Fonseca et al. [10] and Silva et al. [12] for the phenol and flavonoid contents and antioxidant activity may be associated with the presence of lipophilic compounds present in the calli extracts and the hexane fractions of the leaves and stems of *S. brasiliensis*. Phytochemical screening revealed the presence of steroids/terpenes in the hexane fractions of the leaves [10]. Gas chromatography–mass spectrometry (GS-MS) analysis of the hexane fraction of the stems characterized terpenoid compounds, such as neophytadiene (0.34%), phytone (0.94%), kaurene (0.80%), and (*Z*)-phytol (2.32%), and phytosterols, such as lathosterol (1.37%), campesterol (2.13%), and *β*-stigmasterol (4.22%) [12]. The chemical profile by ^1^H NMR analysis suggested an increase in lipophilic compounds for calli extracts, such as triterpenoids and steroid compounds (Figure 5). This corroborates the literature data and can be correlated with the observed antioxidant activity [47,48,49].

Ursane-type triterpenoids isolated from sour jujube (*Ziziphus jujuba*) showed antioxidant activity. The compound 2α,3β,28-trihydroxyurs-20(30)-ene exhibited high antioxidant activity, being 18.9 times greater than ascorbic acid [47]. Phytosterols have attracted attention for their anti-inflammatory, antioxidant, and pro-apoptotic potential, which allows them to decrease inflammation, neutralize free radicals, and induce tumor cell death [48]. Stigmasterol has demonstrated antioxidant potential in numerous studies, both in vitro and in vivo [49]

The antioxidant activity of fatty acids has also been reported. Fratiani et al. [50] determined the fatty acid composition of five cold-pressed plum seed oils and evaluated their antioxidant activity. All tested oils showed antioxidant activity by DPPH, FRAP, and ABTS (2,2′-azinobis(3-ethylbenzothiazoline-6-sulfonic acid) assays. The main fatty acids identified in the oils were oleic acid (39.4–76.3%) and linoleic acid (15.1–46.2%). Boyapati et al. [51] demonstrated the antioxidant activity of pitaya seed oil by DPPH and FRAP assays, with its main components being palmitic, oleic, and linoleic acids. These results indicated that fatty acids are a source of antioxidant agents. Thus, we can suggest that at least in part, the antioxidant potential exhibited by calli extracts may also be related to the unsaturated fatty acids in these samples.

## 4. Materials and Methods

### 4.1. Chemicals

For this study, 2,2-diphenyl-1-picryl-hydrazyl (DPPH), 2,6-di-*tert*-butyl-4-methylphenol (BHT), quercetin, gallic acid, dichlorophenoxyacetic acid (2,4-D), 6-benzylaminopurin (BAP), 2,4,6-tris(2-pyridyl)-s-triazine (TPTZ), methanol-*d*_4,_ and 4-amino-3,5,6-trichloro-2-pyridinecarboxylic acid (picloram) were use and purchased from Sigma-Aldrich (Saint Louis, MO, USA). Folin–Ciocalteu was obtained from Dinâmica Química Contemporânea (Indaiatuba, Brazil). Ethanol was purchased from Alphatec (Pinhais, Brazil). Sodium acetate (Na_2_O_2_CCH_3_),ferric chloride (FeCl_3_), and sucrose were purchased from Synth (Diadema, Brazil). Sodium carbonate (Na_2_CO_3_) and methanol (CH_3_OH) were obtained from Cromato Produtos Químicos (Diadema, Brazil). Captan was acquired from Bayer (São Paulo, Brazil). Pure bacteriological agar powder was obtained from Himedia (Maharashtra, India). Aluminum chloride (AlCl_3_) was obtained from Vetec (Duque de Caxias, Brazil).

### 4.2. Plant Material

**Figure 7:**
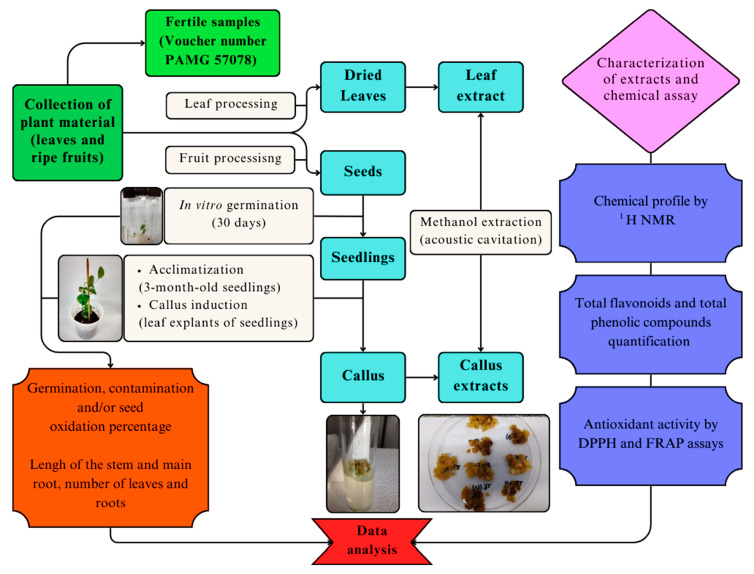
Flowchart of the experimental steps.

*Smilax brasiliensis* leaves and ripe fruits were collected in the Brazilian Cerrado located in Ijaci, South Minas Gerais State, Brazil (21°13′46″ S and 44°55′65″ W, average altitude 908 m) in August 2018 (SISBIO n. 24542-5). Ripe fruits of *S. brasiliensis* were processed manually to remove the seeds. The seeds were treated for 15 min with 5% Captan SC^®^ and stored in a cold room at 4 °C until use. Fertile samples were also collected, and the vouchers were identified by Dr. Regina Helena Potsch Andreata and deposited in the PAMG Herbarium (PAMG 57078) at the Agricultural Research Company of Minas Gerais (EPAMIG). This study has access authorization to the genetic heritage with Access Registration No. A605DF4, granted by SISGEN/CGEN/MMA, for the purpose of research and technological development in accordance with the Brazilian Biodiversity Law (13.123/2015). A flowchart of the experimental steps is shown in Figure 7.

### 4.3. In Vitro Germination

For germination, the seeds were disinfected with 25% sodium hypochlorite for 15 min and then washed with autoclaved distilled water five times. The seeds were mechanically scarified with a scalpel and immersed in autoclaved distilled water for 2 h. One hundred seeds were placed to germinate on MS basal medium with 30 g/L sucrose and solidified with 7 g/L agar. The pH was adjusted to 5.8 ± 0.1 with NaOH 0.1 N, and the medium was sterilized at 120 °C (1.37 × 10^5^ Pa) for 20 min. The seeds were transferred to a growth chamber and kept at 27 ± 2 °C for 30 days, in the presence of light (16:8 h light/dark regime, light intensity of 40 μmol/m^2^ s^1^) provided by fluorescent cool light lamps for 7 months. After 30 days, the germination percentage and whole plant rate were assessed. The presence of contamination and/or oxidation was also evaluated during the experimental stage.

### 4.4. Seedlings Acclimatization

For the acclimatization process, three-month-old seedlings were subjected to the following steps: (1) measuring stem length and counting the number of leaves and roots; (2) washing the roots to remove the culture medium; (3) transfer of seedlings to an ex vitro environment consisting of plastic cups containing vermiculite as substrate; (4) package the seedlings in sealed plastic bags to maintain humidity in the ex vitro environment; (5) gradual transfer of seedlings to natural environment with more sunlight every 7 days; (6) gradual opening of the plastic bags every 3 days, through small holes made in the plastic, to reduce internal humidity and allow the leaves to adapt to the ex vitro environment; (7) completely remove the plastic bag and transfer the seedlings to the ground, in full sun. Before transferring the seedlings to the soil, the length of the stem and main root was measured, and the number of leaves and roots of the acclimatized seedlings was counted.

### 4.5. Callus Induction

The leaf segments (5 × 5 mm) obtained from 7-month-old seedlings were placed on MS basal medium [38], with 30 g/L sucrose and supplemented with PGR in the following concentrations: T0—Control (no PGR); T1—0.5 μg/mL 2.4-D (dichlorophenoxyacetic acid); T2—1 μg/mL 2.4-D; T3—0.5 μg/mL picloram (4-amino-3,5,6-trichloro-2-pyridinecarboxylic acid); T4—1.0 μg/mL picloram; T5—0.5 μg/mL 2,4-D + 0.5 μg/mL BAP (6-benzylaminopurine); T6—0.5 μg/mL picloram + 0.5 μg/mL BAP. The pH was adjusted to 5.8 ± 0.1 with NaOH 0.1 N, and the media were solidified with 7 g/L agar. The media were sterilized at 120 °C for 20 min. The explants were transferred to a growth chamber and kept in the presence of light (in the same conditions described previously) and in the absence of light. The completely randomized experimental design was used and comprised of 40 replicates composed of one test tube, each of which contained an explant, totaling 280 plots. After 12 months in these conditions, the percentage of callus induction was evaluated. Primary calli were subcultured three times, every 8th week, using the same treatments and initial conditions. After this period, the calli were collected, and the consistent, fresh mass (FM), dry mass (DM), and dry mass yield (DMY) of calli were evaluated. To determine the dry mass, calli were dried in an oven (4023D, Nova Ética, Brazil) at 40 °C until mass stabilization. The dry mass yield (%) was obtained by the following formula: (dry mass/fresh mass × 100). The presence of contamination and/or oxidation was also evaluated during this experimental stage.

### 4.6. Obtaining Extracts

The dry calli (1 g) obtained from each treatment were crushed and extracted with 10 mL of methanol (P.A.) by acoustic cavitation in periods of 30 min, totaling 4 h in the process. The material was filtered, and the extract was dried in a rotary evaporator at 35 °C to remove the solvent. The extraction process was repeated two more times. At the end of the process, the extracts obtained from the three extractions for each treatment were combined.

For comparison, 1 g of dried leaves of *S. brasiliensis* obtained from Brazilian Cerrado was also extracted with 10 mL of methanol under the same conditions mentioned above. The solvent was dried in a rotary evaporator, and the extracts obtained from the three extractions were combined, obtaining the methanol extract.

### 4.7. Determination of the Metabolic Profile of Methanol Extracts by Nuclear Magnetic Resonance (NMR)

Samples (15.0 mg) were dissolved in 800 μL of methanol-*d*_4_ containing 0.01% (*w*/*v*) TSP-*d*_4_ (3-(trimethylsilyl)propionic-2,2,3,3-*d*_4_ acid sodium salt), vortexed for 1 min, and placed in an ultrasound bath for 20 min. After centrifugation at 17,000× *g* for 15 min, the supernatants (700 μL) were transferred to 5 mm diameter NMR tubes. The ^1^H NMR experiments were performed in a Bruker Avance Neo 600 MHz, Fällanden, Switzerland and the spectra were acquired at 300 K with a spectral window (SW) of 16 ppm, a number of digitized points (TD) of 64 K, with HDO (deuterated water) signal pre-saturation, acquisition number (NS) of 128, acquisition (AQ), and waiting times before each acquisition (d1) of 3.2 s and 5.0 s, respectively. All spectra were obtained using the zgcppr pulse sequence and processed using a 0.3 Hz line broadening before the Fourier transform. The phases and baselines were automatically corrected using the TopSpin 4.4.0 program, and finally, the spectra were calibrated by the TSP-*d*_4_ signal at 0.00 ppm.

The ^1^H-^1^H COSY (Correlation Spectroscopy) homonuclear correlations were recorded with a standard 90°–90° pulse sequence and field gradient in the *z*-axis direction. These experiments were acquired with AQ(F2) of 192 ms, NS of 4, and d1 of 2.0 s, under SW of 10 ppm in both dimensions, with TD(F1) and TD(F2) equal to 1024 and 2048, respectively. The Fourier transform was applied, and the transformed data were symmetrized. Two-dimensional *J*-Resolved NMR spectra were acquired using the jresgpprqf pulse program, 16 scans per 128 increments in F1 and 2K for F2 using spectral widths of 13,157.895 Hz in F2 and 120 Hz in F1. A 2.0 s relaxation delay was employed. The *J*-Resolved spectra were symmetrized and tilted, and then calibrated.

### 4.8. Determination of the Content of Total Phenolic Compounds and Flavonoids

Total phenolic compounds content was determined by the Folin–Ciocalteu method with some adaptations [52] using 2250 μL of the Folin–Ciocalteu reagent, previously diluted in distilled water (1:4, *v*/*v*), 250 μL of the sample, and 250 μL of 4% sodium carbonate saturated solution, which, after stirring, were allowed to stand for 30 min at room temperature. The absorbance reading was performed in a spectrophotometer (Genesis 10S, Thermo Scientific, Madison, WI, USA) at a wavelength of 750 nm. Gallic acid was used as a reference compound to produce the calibration curve. The determination was performed in triplicate, and the content of phenolic compounds was expressed as μg of gallic acid equivalents/mg of extract (μg GAE/mg).

Total flavonoid content was determined with aluminum chloride with some adaptations [53] using 1900 μL of 50% ethanol, 100 μL of extracts, and 500 μL of AlCl_3_ solution at 5% (*w*/*v*). After 30 min of rest, the absorbance was read at 425 nm in a spectrophotometer (Genesis 10S, Thermo Scientific, Madison, WI, USA). Quercetin was used as a reference compound to produce the calibration curve. The determination was performed in triplicate, and the total flavonoid content was expressed as μg of quercetin equivalents/mg of extract (μg QE/mg).

### 4.9. Assessment of Antioxidant Activity

Antioxidant activity was evaluated by capturing free radicals 2,2-diphenyl-1-picryl-hydrazyl (DPPH) [54], and by reducing iron ion Fe^3+^ to Fe^2+^ (FRAP) [55].

A DPPH solution (0.002% *w*/*v*) was prepared in ethanol. Dilutions were prepared from 1 mg/mL solutions of extracts. A total of 75 μL of the samples was added to the microplate wells, and after that, 150 μL of the DPPH solution was added. Samples were pipetted in triplicate for each of the five concentrations used (1, 10, 100, 250, and 500 μg/mL). BHT (2,6-di-*tert*-butyl-4-methylphenol) was used as a reference compound. A volume of 75 μL of the BHT solution and 150 μL of the DPPH solution was used for the reference test. For sample blanking, 75 μL of test samples or reference compound and 150 μL of ethanol were used. The positive control of the test was prepared by adding 225 μL of the DPPH solution, and for the blank, adding 225 μL of ethanol. The plate was covered and left in the dark at room temperature (25 °C). After 30 min, the absorbance at 517 nm was measured in a spectrophotometer (Power Wave XS2/US, Biotec, Winooski, VT, USA). The percentage of DPPH inhibition was calculated by the following equation: % DPPH inhibition = [1 − (Aa/Ab)] × 100, where Aa = sample absorbance and Ab = absorbance of the DPPH solution. The EC_50_ calculation (effective concentration to decolorize 50% of the DPPH solution) was performed using the probit analysis method.

For the FRAP assay, BHT and extracts were diluted in ethanol and tested at concentrations of 2, 5, 10, 15, and 30 μg/mL. In a 96-well plate, 60 μL of the samples and 240 μL of the reagent containing ferric ions (0.3 M sodium acetate buffer, pH 3.6 + 10 mM TPTZ + 20 mM FeCl_3_) were added; 60 μL of ethanol was pipetted into control wells. The test was performed in triplicate. After 30 min in a dark environment at 25 °C, the plate was read in a spectrophotometer (Power Wave XS2/US, Biotec, Winooski, VT, USA), using a wavelength of 595 nm. The percentage of antioxidant activity was calculated by the equation: % of FRAP = {(Abs sample − Abs control)/Abs sample} × 100, where Abs control = absorbance of FRAP reagent + ethanol and Abs sample = absorbance of FRAP reagent + samples or standard in ethanol. The calculation of the EC_50_ (effective concentration of the samples required to reduce Fe^3+^ ions to Fe^2+^ ions by 50%) was performed using the probit method of analysis.

### 4.10. Statistical Analysis

The data are presented as the mean ± SE. Statistical differences were determined by analysis of variance (ANOVA) followed by Student’s *t*-test using GraphPad Prism 5.0 software. Statistical differences were determined by the Tukey’s Test (*p* ˂ 0.05).

## 5. Conclusions

This study demonstrated promising results for seed germination and the growth of *Smilax brasiliensis* during the acclimatization process, which could lead to greater productivity in the future. The results also contribute to species conservation.

The NMR spectra suggest that the different treatments with PGR may influence calli’s metabolic pathway, changing metabolism in relation to leaves of *S. brasiliensis.* The chemical profile of the calli extracts obtained by ^1^H NMR spectra suggests an increase in lipophilic compound contents, such as common unsaturated fatty acids, triterpenoid or steroid compounds, and amino acids in the calli extracts compared to the leaf extract.

As revealed by DPPH and FRAP assays, the calli extracts exhibited antioxidant activity. Despite showing lower total phenolic compounds and flavonoid levels than the leaf methanol extract, the antioxidant activity of calli extracts can be attributed, at least in part, to lipophilic compounds. These compounds, such as common unsaturated fatty acids, triterpenoids, or steroid compounds, present in these samples could potentially replace synthetic antioxidants in preserving foods, cosmetics, and medicines, sparking new avenues for research and application.

## 6. Patents

The work reported in this manuscript resulted in patent deposits in Brazil. The INPI deposit numbers are BR 10 2023 000,056 8 and BR 10 2023 001,554 9.

## Figures and Tables

**Table 1:** Conditions used for the callus induction were obtained from *Smilax brasiliensis* leaf explants.

Treatments	Plant Growth Regulators (PGR)	Concentration (µg/mL)	Light
T1	2.4-D (dichlorophenoxyacetic acid)	0.5	Absence
T2	2.4-D (dichlorophenoxyacetic acid)	1.0	Absence
T4	Picloram	1.0	Presence
T6	Picloram + BAP (6-benzylaminopurine)	0.5 + 0.5	Absence

**Table 2:** Callus induction (CI), consistency, fresh mass (FM), dry mass (DM), and dry mass yield (DMY) of the calli obtained from *Smilax brasiliensis* leaf explants.

Treatments	CI (%)	Consistency	FM (g)	DM (g)	DMY (%)
T1	50%	friable	1.3239 ± 0.5113 ^ab^	0.1797 ± 0.1298 ^a^	13.57 ^b^
T2	25%	friable	0.7901 ± 0.3020 ^b^	0.2411 ± 0.0773 ^a^	30.05 ^a^
T4	75%	friable	2.3656 ± 0.8435 ^a^	0.1361 ± 0.0469 ^a^	5.75 ^c^
T6	100%	friable	2.5730 ± 1.3246 ^a^	0.1232 ± 0.0720 ^a^	4.79 ^c^

The results are means ± SE (n = 5) for FM and DM. Means followed by the same letter in the same column do not differ according to the Tukey test (*p* ˂ 0.05).

**Table 3:** Weights and dry material yields % for each extraction obtained from the callus of *Smilax brasiliensis* (T1, T2, T4, and T6) and its leaves (ME).

Samples	T1	T2	T4	T6	ME
Mass 1 (g)	0.1210	0.0760	0.0950	0.0650	0.0950
Yield 1 (%)	12.10	7.60	9.50	6.50	9.50
Mass 2 (g)	0.0994	0.0482	0.0522	0.0215	0.0646
Yield 2 (%)	9.94	4.82	5.22	2.15	6.46
Mass 3 (g)	0.0345	0.0189	0.0169	0.0113	0.0319
Yield 3 (%)	3.45	1.89	1.69	1.13	3.19
Final Mass (g)	0.2549	0.1431	0.1641	0.0978	0.1915
Final Yield (%)	25.49	14.31	16.41	9.78	19.15

T1: 0.5 µg/mL 2.4-D (dichlorophenoxyacetic acid), T2: 1 µg/mL 2.4-D, T4: 1.0 µg/mL picloram, T6: 0.5 µg/mL picloram + 0.5 µg/mL BAP (6-benzylaminopurine), ME: leaf methanol extract.

**Table 4:** Total phenolic (TCP) and flavonoid (FC) content, DPPH and FRAP IC_50_ values for dry calli and leaf extracts obtained from *Smilax brasiliensis*.

Samples	TCP (μg GAE/mg) ^1^	FC (μg QE/mg) ^2^	DPPH IC_50_ (μg/mL) ^3^	FRAP IC_50_ (μg/mL) ^4^
T1	27.81 ± 0.07 ^c^	15.41 ± 0.02 ^b^	178.69 ± 32.87 ^cd^	25.38 ± 3.26 ^c^
T2	36.36 ± 0.04 ^b^	13.14 ± 0.02 ^b^	235.44 ± 38.77 ^d^	33.97 ± 5.32 ^c^
T4	33.73 ± 0.04 ^b^	8.08 ± 0.01 ^c^	135.13 ± 10.31 ^c^	12.26 ± 1.88 ^b^
T6	25.32 ± 0.03 ^c^	5.83 ± 0.01 ^c^	142.56 ± 19.63 ^c^	14.14 ± 1.75 ^b^
ME	186.73 ± 6.60 ^a^	26.67 ± 0.33 ^a^	6.59 ± 0.87 ^a^	0.88 ± 0.12 ^a^
BHT	-	-	16.36 ± 1.63 ^b^	1.47 ± 0.18 ^a^

T1: 0.5 μg/mL 2.4-D (dichlorophenoxyacetic acid), T2: 1 μg/mL 2.4-D, T4: 1.0 μg/mL picloram, T6: 0.5 μg/mL picloram + 0.5 μg/mL BAP (6-benzylaminopurine), ME: leaf methanol extract, BHT: 2,6-di-*tert*-butyl-4-methylphenol. ^1^ TCP: μg of gallic acid equivalents/mg of extract (μg GAE/mg). ^2^ FC: μg of quercetin equivalents/mg of extract (μg QE/mg). ^3^ IC_50_: concentration (in μg/mL) of samples required to inhibit the formation of DPPH radicals by 50%. ^4^ IC_50_: concentration (in μg/mL) of samples required to reduce [Fe(III) (2,4,6-tripyridyl-s-triazine)]^3+^ by 50%. The results are means ± SE (n = 3). Means followed by the same letter in the same column do not differ according to the Tukey’s Test (*p* ˂ 0.05).

**Table 5:** Antioxidant activity by DPPH and FRAP methods for dry calli and leaf extracts obtained from *Smilax brasiliensis*.

**Samples**	**DPPH Free Radical Capture (%)**				
	1 μg/mL	10 μg/mL	100 μg/mL	250 μg/mL	500 μg/mL
T1	36.56 ± 0.38 ^a^	37.67 ± 0.79 ^b^	42.89 ± 0.36 ^c^	46.71 ± 0.38 ^c^	74.59 ± 0.71 ^b^
T2	36.17 ± 0.42 ^a^	36.85 ± 0.25 ^b^	41.54 ± 0.08 ^c^	45.60 ± 0.08 ^c^	62.31 ± 0.79 ^c^
T4	36.61 ± 0.55 ^a^	37.62 ± 0.17 ^b^	46.13 ± 0.25 ^c^	53.86 ± 0.51 ^b^	92.61 ± 1.31 ^a^
T6	36.69 ± 0.54 ^a^	37.11 ± 0.08 ^b^	44.53 ± 0.87 ^c^	56.38 ± 0.79 ^b^	65.42 ± 1.08 ^c^
ME	34.05 ± 0.63 ^a^	57.53 ± 0.38 ^a^	96.28 ± 0.08 ^a^	97.00 ± 0.51 ^a^	98.16 ± 0.84 ^a^
BHT	18.50 ± 0.65 ^b^	25.90 ± 0.64 ^c^	86.00 ± 0.56 ^b^	91.40 ± 0.28 ^a^	94.02 ± 0.51 ^a^
**Samples**	**FRAP activity (%)**				
	2 μg/mL	5 μg/mL	10 μg/mL	15 μg/mL	30 μg/mL
T1	0.00 ^d^	3.99 ± 0.55 ^c^	21.63 ± 2.55 ^c^	34.89 ± 1.17 ^c^	55.40 ± 3.89 ^c^
T2	0.00 ^d^	2.08 ± 0.87 ^c^	16.03 ± 4.14 ^c^	26.60 ± 3.33 ^c^	44.52 ± 1.58 ^d^
T4	9.29 ± 3.21 ^c^	26.15 ± 0.92 ^b^	43.50 ± 1.99 ^b^	57.77 ± 6.75 ^b^	70.02 ± 0.55 ^b^
T6	0.00 ^d^	20.57 ± 0.62 ^b^	40.07 ± 0.89 ^b^	51.80 ± 1.04 ^b^	69.21 ± 0.51 ^b^
ME	74.79 ± 0.24 ^a^	88.72 ± 0.22 ^a^	93.11 ± 1.09 ^a^	95.64 ± 0.17 ^a^	97.55 ± 0.02 ^a^
BHT	59.37 ± 0.10 ^b^	81.60 ± 0.33 ^a^	89.32 ± 0.36 ^a^	92.43 ± 0.17 ^a^	94.97 ± 0.06 ^a^

T1: 0.5 μg/mL 2.4-D (dichlorophenoxyacetic acid), T2: 1 μg/mL 2.4-D, T4: 1.0 μg/mL picloram, T6: 0.5 μg/mL picloram + 0.5 μg/mL BAP (6-benzylaminopurine), ME: leaf methanol extract, BHT: 2,6-di-*tert*-butyl-4-methylphenol. The results are means ± SE (n = 3). Means followed by the same letter in the same column do not differ according to the Tukey’s Test (*p* ˂ 0.05).

## Data Availability

Data are contained within the article.

## Supplementary Materials

The following supporting information can be downloaded at https://www.mdpi.com/article/10.3390/plants14091383/s1, Figure S1: HSQC contour map of methanol extract from calli obtained from *S. brasiliensis* grown under Treatment 6 (0.5 μg/mL picloram + 0.5 μg/mL BAP) (600 MHz, methanol-*d*_4_ containing 0.01% (*w*/*v*) TSP-*d*_4_), in the region of 5.6 to 0.6 ppm for ^1^H NMR and 10–135 ppm for ^13^C-NMR.; Figure S2: COSY contour map of methanol extract from calli obtained from *S. brasiliensis* grown under Treatment 6 (0.5 μg/mL picloram + 0.5 μg/mL BAP) (600 MHz, methanol-*d_4_* containing 0.01% (*w*/*v*) TSP-*d*_4_), in the region of 5.4 to 0.8 ppm.
