# Supplementary material for: In Vitro Production of Smilax brasiliensis Seedlings, Callus Induction, Chemical Profile, and Assessment of Antioxidant Activity
Source: Plants (Basel). 2025 May 3;14(9):1383. doi: 10.3390/plants14091383 (PMC12074387; doi:10.3390/plants14091383)
Supplement: Supplementary file 1 [file plants-14-01383-s001.zip › plants-3562269-supplementary.pdf]

Supplementary Materials

In vitro Production of *Smilax brasiliensis* Seedlings, Callus Induction, Chemical Profile, and Assessment of Antioxidant Activity

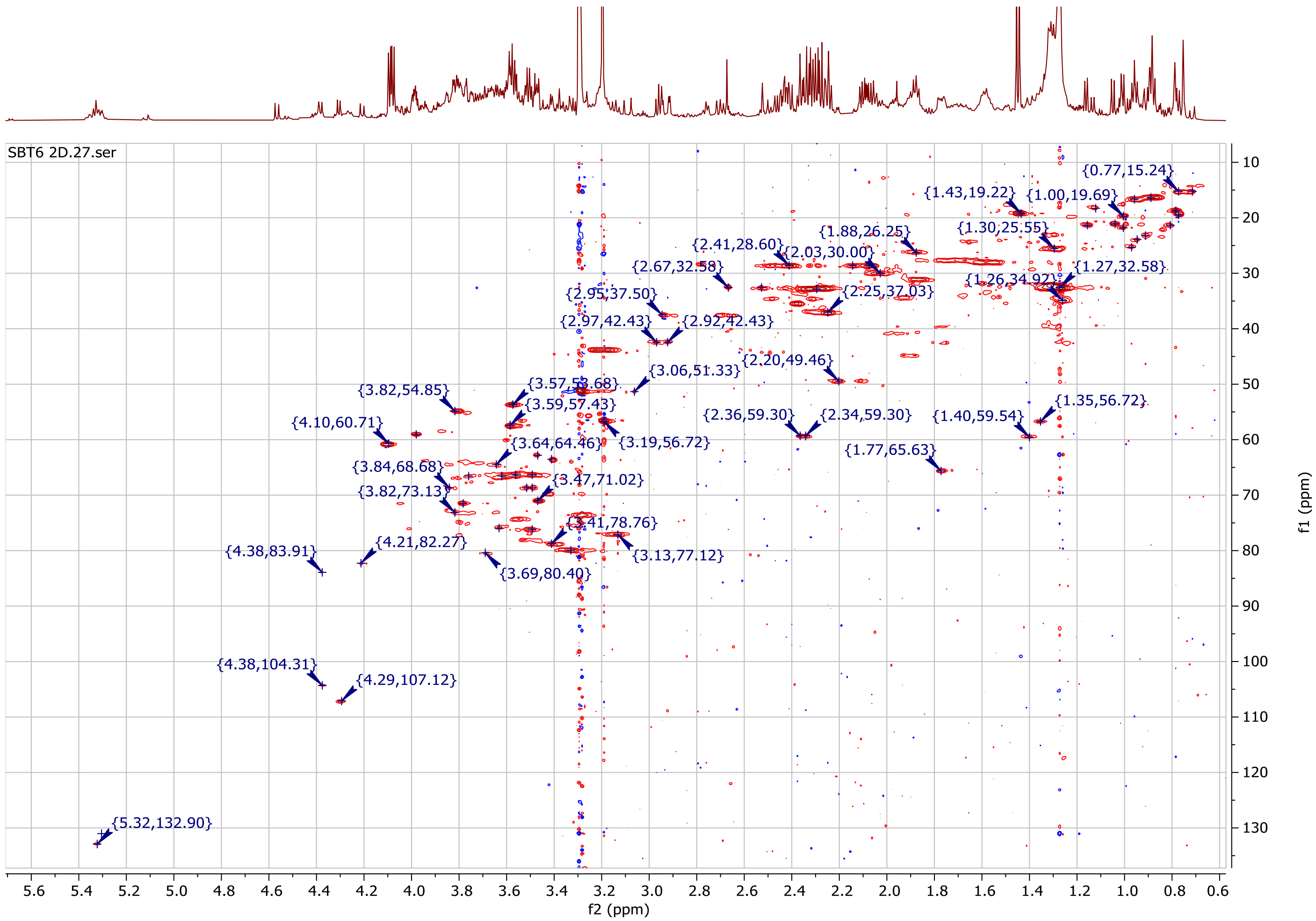

Figure S1: HSQC contour map of methanol extract from calli obtained from *S. brasiliensis* grown under Treatment 6 (0.5 µg/mL picloram + 0.5 µg/mL BAP) (600 MHz, methanol-d_4 containing 0,01 % (w/v) TSP-d_4), in the region of 5.6 to 0.6 ppm for ^{1}H NMR and 10-135 ppm for ^{13}C-NMR.

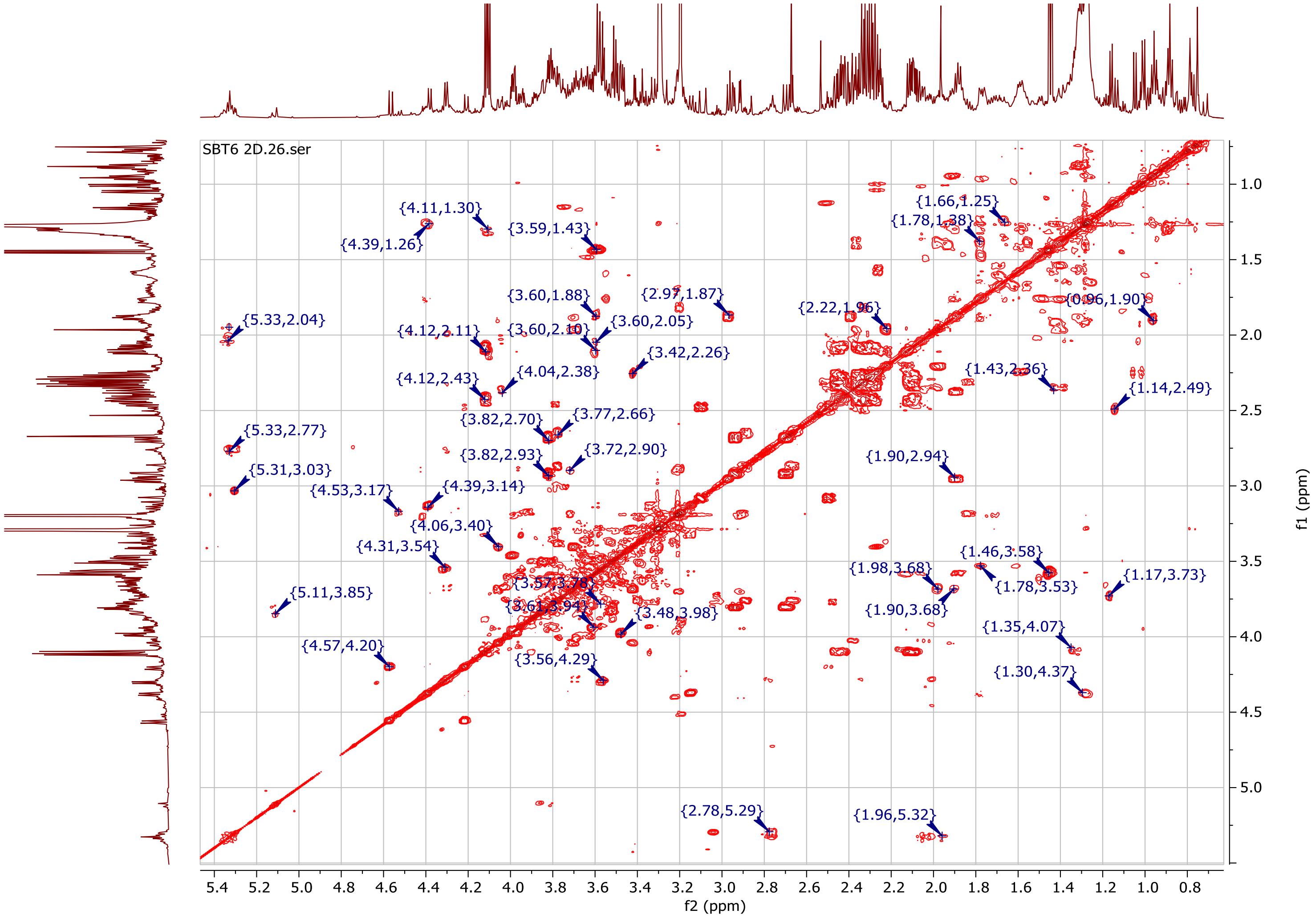

Figure S2: COSY contour map of methanol extract from calli obtained from *S. brasiliensis* grown under Treatment 6 (0.5 µg/mL picloram + 0.5 µg/mL BAP) (600 MHz, methanol-d_4 containing 0,01 % (w/v) TSP-d_4), in the region of 5.4 to 0.8 ppm.
